# Exploring epistasis in candidate genes for rheumatoid arthritis

**DOI:** 10.1186/1753-6561-1-s1-s70

**Published:** 2007-12-18

**Authors:** Marylyn D Ritchie, Jacquelaine Bartlett, William S Bush, Todd L Edwards, Alison A Motsinger, Eric S Torstenson

**Affiliations:** 1 Department of Molecular Physiology and Biophysics, Center for Human Genetics Research, Vanderbilt University Medical Center, 21st Avenue South and Garland Avenue, Nashville, Tennessee 37232, USA; 2 Department of Statistics, North Carolina State University, 2501 Founders Drive, Campus Box 8203, Raleigh, North Carolina 27695, USA

## Abstract

The identification of susceptibility genes for common, chronic disease presents great challenges. The development of novel statistical and computational methodologies to help identify these genes is an area of great necessity. Much research is ongoing and the Genetic Analysis Workshop (GAW) is a venue for the dissemination and comparison of many of these methods. GAW15 included real data sets to look for disease susceptibility genes for rheumatoid arthritis (RA). RA is a complex, chronic inflammatory disease with several replicated disease genes, but much of the genetic variation in the phenotype remains unexplained. We applied two computational methods, namely multifactor dimensionality reduction (MDR) and grammatical evolution neural networks (GENN), to three data sets from GAW15. While these analytic methods were applied with the intention of detecting of multilocus models of association, both methods identified a strong single locus effect of a single-nucleotide polymorphism (SNP) in *PTPN22 *that is significantly associated with RA. This SNP has previously been associated with RA in several other published studies. These results demonstrate that both MDR and GENN are capable of identifying a single-locus main effect, in addition to multilocus models of association. This is the first published comparison of the two methods. Because GENN employs an evolutionary computation search strategy in comparison to the exhaustive search strategy of MDR, it is encouraging that the two methods produced similar results. This comparison should be extended in future studies with both simulated and real data.

## Background

Rheumatoid arthritis (RA) is a complex, chronic inflammatory disease affecting approximately 1% of the population [1]. It is hypothesized that risk for RA is due to both genetic and environmental contributions; however, the etiology of the disease remains unknown [2]. Many epidemiological studies have been performed to investigate the genetics of RA. Oliver et al. [3] reviewed articles published between October 2004 and November 2005 and found that in addition to the *HLA-DRB1 *gene, association of *PTPN22 *with RA has been consistently replicated in numerous studies. The genetics of RA are beginning to be unraveled, but the variants discovered do not account for all of the genetic variation in RA. These and other successes in genetic research of common, complex disease contribute to optimism that contemporary study design philosophy is adequate for these investigations, and must simply be scaled to detect the smaller effects that contribute to these diseases.

Many common, complex diseases are currently being investigated and a recurrent theme emerges: complex diseases are likely the result of many genetic and environmental factors. Identifying all polymorphisms that present an increased risk of disease is difficult. Epistasis, or gene × gene interaction, is increasingly assumed to play a crucial role in the genotype-to-phenotype relationship of common diseases [4-6]. Unfortunately, the detection of gene × gene and gene × environment interactions requires large samples due to the dimensionality of evaluating combinations of multiple variables. This phenomenon is referred to as the curse of dimensionality [7]; that is, as the number of genetic or environmental factors increases, the number of possible interactions increases exponentially and many contingency table cells will have little or no data.

To deal with this issue, much research is needed for improved statistical methodologies. In this study, we will apply two computational approaches to explore gene × gene interactions associated with RA: multifactor dimensionality reduction (MDR) and grammatical evolution neural network (GENN). The goals of this study are as follows: 1) to identify genes associated with RA; 2) to compare the results of an exhaustive search strategy (MDR) and an evolutionary computation search strategy (GENN), and 3) to demonstrate alterative fitness metrics for MDR. We will demonstrate that both MDR and GENN detected a strong single locus effect of *PTPN22*; no multi-locus models were identified. This result supports the hypotheses that: 1) *PTPN22 *is associated with RA and 2) MDR and GENN can both detect single-locus effects.

## Methods

### Sample

In this study, we are using three case-control data sets as part of GAW15. Data set 1 is a candidate gene study exploring 14 SNPs in *PTPN22 *from Carlton et al. [8]. This data set has 1269 cases (some of which are affected sibling pairs) and 1519 unrelated controls. Data set 2 is a candidate gene study exploring 20 SNPs in several candidate genes including *PTPN22*, *CTLA4*, *TNFRS1*, and *PADI4 *from Plenge et al. [2]. This data set includes 839 cases (including affected sibling pairs) and 855 unrelated controls. Finally, data set 3 is a dense panel of 2300 SNPs genotyped by Illumina for a 10-kb region of chromosome 18 that has demonstrated evidence for linkage in both US and French whole-genome screens. This data set included 460 cases and 460 controls. We treated these data sets as three independent sets for all analyses.

### Computational methods

Multifactor dimensionality reduction (MDR) is a data reduction method for detecting multi-locus genotype combinations that predict disease risk for common, complex disease. MDR pools genotypes into "high-risk" and "low-risk" groups in order to reduce multidimensional data into only one dimension. MDR has been described in detail previously [9-11].

Three alternative fitness measures were used for comparing multilocus models in MDR: balanced accuracy (BA), model-adjusted balanced accuracy, and normalized mutual information (NMI). BA weighs the classification accuracy of the two classes equally and it is thought to be more powerful than using accuracy alone when data are imbalanced, or when the counts of cases and controls are not equal [12,13]. BA is calculated from a 2 × 2 table relating exposure to status by [(sensitivity+specificity)/2]. Model-adjusted balanced accuracy is similar, but uses a different threshold in the MDR modeling that is based on the actual counts of case and control samples in the data being evaluated. In a given combination of loci, individuals may have missing data at any one locus or multiple loci. The MDR threshold can be adjusted to take into account the precise number of individuals with complete data for that particular multilocus combination. We performed this threshold adjustment in combination with balanced accuracy (model-adjusted BA). Normalized mutual information (NMI) is a measure of information transmission based on Shannon's entropy. It has been described by Forbes as a performance measure for classifiers. The NMI is the proportion of information provided by the algorithm's classification, which is contained in the true status or outcome [14]. For an error-free classifier, NMI = 1, because the algorithm's classification and the status are identical.

Grammatical evolution neural network (GENN) is a novel pattern recognition method developed to detect main effects or multilocus models of association without exhaustively searching all possible multilocus combinations. Grammatical evolution (GE) is a machine-learning algorithm inspired by the biological process of transcription and translation. GE uses a genetic algorithm in combination with a prespecified grammar (set of translation rules) to automatically evolve an optimal computer program. GENN utilizes GE to evolve the inputs (predictor variables), architecture (arrangement of layers and functions), and weights of a neural network (NN) to optimally classify a given dataset.

GENN has been described in detail [15,16]. Briefly, GENN begins with the initialization of parameters specified in the configuration file, including mutation rate, crossover rate, and number of generations. Next, the data are divided into 10 equal parts for 10-fold cross-validation (CV) to evaluate the predictive ability of the models developed. Third, an initial population of random solutions is generated to begin the training process. Fourth, each individual genome is translated into a NN according to the rules of the grammar. Each NN is evaluated on the training set and its fitness is recorded. Fifth, the best solutions are selected for crossover and reproduction using user-specified proportions. The new generation (created by a selection technique specified in the configuration file) begins the cycle again. This continues until some stopping criterion is met, a balanced classification accuracy of 100% is found, or a limit on the number of generations is reached. An optimal solution is identified after each generation. At the end of GENN evolution, the overall best solution is selected as the optimal NN. Sixth, this best GENN model is tested on the 1/10 of the data left out to estimate the prediction accuracy of the model. Steps two through six are performed ten times using a different 9/10 of the data for training and 1/10 of the data for testing. GENN has been shown to have high power to detect a range of epistatic interactions in simulated data [15,16].

### Data analysis

**Figure 1:**
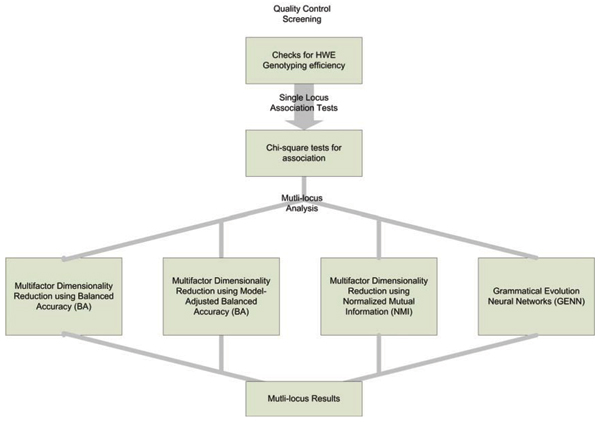
Data analysis plan.

We performed an initial screen of all SNPs testing for Hardy-Weinberg equilibrium (HWE) (separately in cases and controls), genotyping efficiency, and single-locus allelic chi-squared tests for association. Next, we explored patterns of linkage disequilibrium (LD) in each of the three data sets individually to fully understand the correlations between the SNPs prior to analysis. This is essential when conducting analyses where cross-validation is performed and consistency between models will be evaluated. It is conceivable that the algorithm might identify different SNPs in the best model for each CV interval that are actually in LD with one another. If this LD information is not known, it will appear as different association signals. However, if it is known that the SNPs are in LD, one can assume that perhaps it is one signal for that gene region. Finally, we conducted MDR and GENN analyses on all three data sets: MDR with balanced accuracy, MDR with model-adjusted balanced accuracy, MDR with NMI, and GENN. Figure 1 shows our data analysis plan. MDR and GENN assume that all individuals are unrelated. All of our controls were from an independent population but some of the cases were related. We selected one case from each family to create a subset of unrelated cases to use in our analysis (no dupes data set). We also ran the analysis with the all possible case-control pairs using the related cases paired with the unrelated controls (dupes dataset). We ran the analyses both ways, as reported in Table 1.

**Table 1:** Results of MDR and GENN analysis in data sets 1 and 2

		Data set 1		Data set 2	
			
		Dupes	No dupes	Dupes	No dupes
MDR BA	Best model	rs2476601	rs2476601	rs2476601	rs2476601
	BPA	57.63	66.18	58.55	66.28
	CVC (5)	5	5	5	5
	*p*-value	0.01	0.03	0.00	0.00
					
MDR model-adjusted BA	Best model	rs2476601	rs2476601	rs2476601	rs2476601
	BPA	57.99	66.18	58.55	66.28
	CVC (5)	5	5	5	5
	*p*-value	0.01	0.03	0.00	0.00
					
GENN	Best model	rs2476601	rs2476601	rs2476601	rs2476601
	BPA	59.98	58.15	55.93	56.07
	CVC (10)	10	8	10	10
	*p*-value	<0.05	<0.03	<0.05	<0.05
					
MDR NMI	Best model	rs2476601	rs2476601	rs2476601	rs2476601
	NMI	0.03211	0.03528	0.03291	0.02579
	CVC (5)	5	5	5	5
	*p*-value	0.004	0.017	0.0000	0.0000

For these MDR analyses, we conducted five-fold cross-validation and performed 1000 permutations to determine statistical significance. We used several fitness functions to perform model selection as described above. The configuration parameter settings used in the GENN analysis were as follows: 10 demes, migration every 25 generations, population size of 200 per deme, crossover rate of 0.9, and a reproduction rate of 0.1. The algorithm was run for two times the number of generations as variables included in the data set. These parameter settings have been shown to optimize the performance of the GENN method in previous studies [16].

## Results

In the tests for HWE, we found two SNPs out of HWE at *p *< 0.05 in Data set 1 (in cases only), one SNP in Data set 2 (in controls only *p *= 0.045), and 75 SNPs in Data set 3 in controls, 101 SNPs in cases, and 115 SNPs in both. We did not remove any markers from analyses of Data sets 1 or 2 based on HWE. While traditionally deviations from HWE have been interpreted as the result of genotyping error, deviation might actually be the result of important genotype × phenotype association when these deviations occur in cases or controls alone. We did, however, remove 115 markers (all with HWE *p *< 0.05 in cases and controls) from Data set 3. The genotyping efficiency was <95% for 1 marker in Data set 1, 0 markers in Data set 2, and 11 markers in Data set 3. We removed these markers from subsequent analyses. In single locus allelic chi-squared tests for association, we found 9 SNPs statistically significant at *p *< 0.05 in Data set 1, 6 SNPs in Data set 2, and 132 SNPs in Data set 3. All three of these exceed the number expected by chance using α = 0.05. One SNP in *PTPN22*, rs2476601, was highly associated (*p *< 0.001) in both Data set 1 and Data set 2.

For the MDR and GENN analyses, several statistically significant models were detected (shown in Table 1 for Data sets 1 and 2). We report the prediction accuracy and the cross-validation consistency (CVC) for all of the models. For MDR, 5-fold CV was performed and for GENN, 10-fold CV was performed. Thus, the maximum CVC values of 5 and 10 are shown in parentheses Table 1. In dataset 1, MDR with BA, MDR with model-adjusted BA, MDR with NMI, and GENN detected rs2476601 as the best model with statistically significant prediction accuracy of ~57–60% (*p *< 0.05) in the dupes data set and ~58–66% (*p *< 0.03) in the no dupes data set. In Data set 2, MDR with BA, MDR with model-adjusted BA, MDR with NMI, and GENN detected rs2476601 as the best model with statistically significant prediction accuracy (*p *< 0.05). In Data set 3, MDR with BA detected SNP_85 as the best model with a statistically significant prediction accuracy (*p *= 0.02) (not shown). MDR with NMI and GENN did not detect any statistically significant loci in Data set 3.

## Discussion

In this study, we applied several computational approaches for detection of single-locus associations and multilocus models of association to case-control data sets for rheumatoid arthritis. Data sets 1 and 2 replicated the association of rs2476601 using all computational approaches. All case and control samples in Data set 2 are also included in Data set 1, so the replication is not surprising. The minor allele of R620W (rs2476601) is a missense SNP in the hematopoietic-specific protein tyrosine phosphatase gene, *PTPN22*, and has been associated with multiple autoimmune diseases, including RA [8]. This SNP was previously reported for Data sets 1 and 2. This is encouraging validation of these novel methods of analysis.

From a methodological perspective, it is of note that GENN and MDR detected the same main-effect model in both Data sets 1 and 2. This study represents the first published side-by-side comparison of the two methods. Because GENN employs an evolutionary computation search strategy in comparison to the exhaustive search strategy of MDR, it is an important result that the two methods produced similar results. This comparison should be extended in future studies with both simulated and real data. In addition, this is the first evaluation of the model-adjusted balanced accuracy fitness measure for MDR. This will need further validation in future simulation studies.

As mentioned in the Methods section, we performed these analyses in two ways. First, we ran the analysis using one case per family and the unrelated controls (no dupes). Second, we created all possible case-control pairs using the affected sibling pairs and the unrelated controls (dupes). The same loci were detected in all analyses despite these different configurations of the data. However, the prediction accuracy results were slightly different. It is important to note that using the affected siblings and treating them as unrelated may bias the prediction accuracy estimates. While this approach is often done to maximize the use of all samples collected, and fortunately the loci detected did not change, the estimates of the predictive ability should be taken with caution.

## Conclusion

An important conclusion of this study is that while MDR and GENN were applied to these data primarily for the purpose of detecting multilocus models of association, they were successful in detecting statistically significant main effects. This is a key discovery because there has been skepticism in the literature as to whether or not these approaches for detecting epistasis could also be applied to data sets in which main effects are suspected. Thus, if it is unknown whether main effects or interactions are likely to be associated with the phenotype of interest, MDR or GENN could be applied in addition to the traditional statistical approaches and both main effects and interactions could be found.

## Competing interests

The author(s) declare that they have no competing interests.

## Authors' contributions

JB, WSB, AAM, TLE performed all analyses, EST did all data formatting and programming, and all authors contributed to manuscript.

## References

[B1] Ikari K, Kuwahara M, Nakamura T, Momohara S, Hara M, Yamanaka H, Tomatsu T, Kamatani N (2005). Association between *PADI4 *and rheumatoid arthritis: a replication study. Arthritis Rheum.

[B2] Plenge RM, Padyukov L, Remmers EF, Purcell S, Lee AT, Karlson EW, Wolfe F, Kastner DL, Alfredsson L, Altshuler D, Gregersen PK, Klareskog L, Rioux JD (2005). Replication of putative candidate-gene associations with rheumatoid arthritis in >4,000 samples from North America and Sweden: association of susceptibility with *PTPN22*, *CTLA4*, and *PADI4*. Am J Hum Genet.

[B3] Oliver JE, Worthington J, Silman AJ (2006). Genetic epidemiology of rheumatoid arthritis. Curr Opin Rheumatol.

[B4] Moore JH (2003). The ubiquitous nature of epistasis in determining susceptibility to common human diseases. Hum Hered.

[B5] Sing CF, Stengard JH, Kardia SL (2004). Dynamic relationships between the genome and exposures to environments as causes of common human diseases. World Rev Nutr Diet.

[B6] Thornton-Wells TA, Moore JH, Haines JL (2004). Genetics, statistics and human disease: analytical retooling for complexity. Trends Genet.

[B7] Bellman R (1961). Adaptive Control Processes.

[B8] Carlton VE, Hu X, Chokkalingam AP, Schrodi SJ, Brandon R, Alexander HC, Chang M, Catanese JJ, Leong DU, Ardlie KG, Kastner DL, Seldin MF, Criswell LA, Gregersen PK, Beasley E, Thomson G, Amos CI, Begovich AB (2005). *PTPN22 *genetic variation: evidence for multiple variants associated with rheumatoid arthritis. Am J Hum Genet.

[B9] Ritchie MD, Hahn LW, Roodi N, Bailey LR, Dupont WD, Parl FF, Moore JH (2001). Multifactor-dimensionality reduction reveals high-order interactions among estrogen-metabolism genes in sporadic breast cancer. Am J Hum Genet.

[B10] Ritchie MD, Hahn LW, Moore JH (2003). Power of multifactor dimensionality reduction for detecting gene-gene interactions in the presence of genotyping error, missing data, phenocopy, and genetic heterogeneity. Genet Epidemiol.

[B11] Ritchie MD, Motsinger AA (2005). Multifactor dimensionality reduction for detecting gene-gene and gene-environment interactions in pharmacogenomics studies. Pharmacogenomics.

[B12] Mower JP (2005). PREP-Mt: predictive RNA editor for plant mitochondrial genes. BMC Bioinformatics.

[B13] Velez DR, White BC, Motsinger AA, Bush WS, Ritchie MD, Williams SM, Moore JH (2007). A balanced accuracy function for epistasis modeling in imbalanced datasets using multifactor dimensionality reduction. Genet Epidemiol.

[B14] Forbes AD (1995). Classification-algorithm evaluation: five performance measures based on confusion matrices. J Clin Monit.

[B15] Motsinger AA, Dudek SM, Hahn LW, Ritchie MD (2006). Comparison of neural network optimization approaches for studies of human genetics. Lect Notes Comput Sci.

[B16] Motsinger AA, Reif DM, Dudek SM, Ritchie MD (2006). Dissecting the evolutionary process of grammatical evolution optimized neural networks. IEEE Symp Comput Intell Bioinformatics Comput Biol.

